# Risk factors associated with adverse reactions to antituberculosis drugs*

**DOI:** 10.1590/S1806-37132015000100010

**Published:** 2015

**Authors:** Laíse Soares Oliveira Resende, Edson Theodoro dos Santos-Neto

**Affiliations:** Espírito Santo State Court of Justice, Vitória, Brazil. Espírito Santo State Court of Justice, Vitória, Brazil; Federal University of Espírito Santo, Vitória, Brazil. Federal University of Espírito Santo, Vitória, Brazil

**Keywords:** Tuberculosis, Drug-related side effects and adverse reactions, Antitubercular agents, Review

## Abstract

This review sought to identify the available scientific evidence on risk factors associated with adverse reactions to antituberculosis drugs. We performed a systematic review of studies published in the 1965-2012 period and indexed in the MEDLINE and LILACS databases. A total of 1,389 articles were initially selected. After reading their abstracts, we selected 85 studies. Of those 85 studies, 16 were included in the review. Risk factors for adverse reactions to antituberculosis drugs included age > 60 years, treatment regimens, alcoholism, anemia, and HIV co-infection, as well as sodium, iron, and albumin deficiency. Protective factors against hepatic adverse effects of antituberculosis drugs included being male (combined OR = 0.38; 95% CI: 0.20-0.72) and showing a rapid/intermediate N-acetyltransferase 2 acetylator phenotype (combined OR = 0.41; 95% CI: 0.18-0.90). There is evidence to support the need for management of adverse reactions to antituberculosis drugs at public health care facilities.

## Abstract (pt)

Esta revisão buscou identificar a evidência científica disponível sobre os fatores de risco associados às reações adversas a medicamentos (RAM) antituberculose. Foi realizada uma revisão sistemática de estudos publicados entre 1965 a 2012 e indexados nas bases de dados MEDLINE e LILACS. Foram inicialmente selecionados 1.389 artigos. Após a leitura dos resumos, foram selecionados 85 estudos. Dos 85 estudos, 16 foram incluídos na revisão. Os fatores de risco de RAM foram idade > 60 anos, esquemas de tratamento, alcoolismo, anemia, coinfecção pelo HIV e deficiência de sódio, ferro e albumina. Os fatores de proteção contra RAM hepáticas foram o sexo masculino (OR combinada = 0,38; IC95%: 0,20-0,72) e o fenótipo acetilador rápido/intermediário da N-acetiltransferase 2 (OR combinada = 0,41; IC95%: 0,18-0,90). Há, portanto, evidências para subsidiar o manejo de RAM antituberculose nos serviços de saúde pública.

## Introduction

There have been few technological advances in the treatment of tuberculosis (TB). First-line antituberculosis drugs (rifampin, isoniazid, pyrazinamide, and ethambutol) constitute the main therapeutic strategy to control the disease, their efficacy being greater than 95% in susceptible patients.^(^ 1 ^)^

The treatment regimen currently used in developing countries is a fixed-dose, single-tablet combination of four drugs (rifampin, isoniazid, pyrazinamide, and ethambutol) in the intensive phase of treatment in order to reduce primary resistance to the isoniazid-rifampin combination and improve patient adherence to treatment.^(^ 1 ^,^ 2 ^)^ However, treatment discontinuation and dropout persist and result in increased morbidity and mortality from TB.^(^ 3 ^,^ 4 ^)^

Negative outcomes of TB treatment pose risks to individual and public health, prolonging the infection and thus increasing the possibility of transmission of multidrug-resistant bacilli. Therefore, factors associated with treatment failure have been studied in order to improve treatment and prognosis.^(^ 5 ^)^

Adverse drug reactions (ADRs) are defined by the World Health Organization.^(^ 6 ^)^ The Brazilian National Ministry of Health divides adverse reactions to antituberculosis drugs into two large groups on the basis of their severity. Minor adverse effects occur in 5-20% of cases and are thus classified because they require no immediate modification of the standard regimen and, in most cases, call for measures that can be taken at primary care clinics. Major adverse effects are less common; that is, they occur in 3-8% of cases and call for treatment discontinuation or modification, as well as specialized care.^(^ 1 ^,^ 7 ^)^

According to the Brazilian guidelines on TB, adverse reactions to antituberculosis drugs are multifactorial. However, the major determinants of adverse reactions to antituberculosis drugs are the doses, the time of day at which the drugs are administered, age (from the fourth decade of life onward), nutritional status (body weight loss > 15%), alcohol consumption (daily alcohol intake > 101 mL), liver function, kidney function, and co-infection with HIV.^(^ 1 ^,^ 8 ^,^ 9 ^)^

In recent decades, there has been an increasing concern over patient adherence to antituberculosis treatment.^(^ 10 ^)^ Therefore, studies on this topic are warranted, given that adverse reactions during TB treatment are a major factor for treatment nonadherence. The objective of the present study was to identify scientific evidence on risk factors associated with adverse reactions to antituberculosis drugs. 

## Methods

**Chart 1 -:**
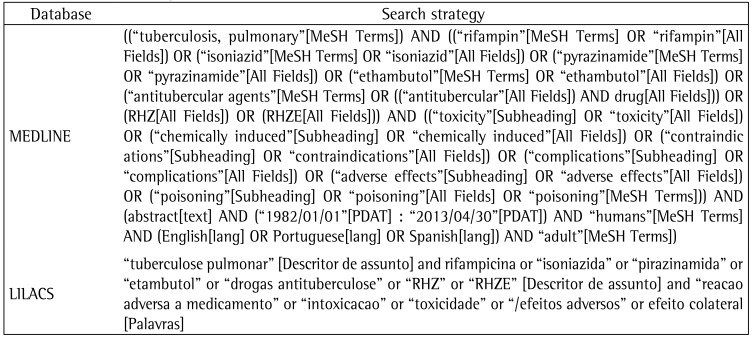
Search strategies used in order to retrieve articles from the databases.

This was a systematic review of studies retrieved from electronic databases and examining risk factors associated with adverse reactions to antituberculosis drugs. The LILACS database was searched for articles published between January of 1982 and April of 2013, and the MEDLINE database was searched for articles published between January of 1965 and April of 2013. The MEDLINE and LILACS databases were searched with the use of MeSH and DeCS terms, respectively, in combination with the appropriate Boolean operators (OR, AND, and NOT), as shown in Chart 1. 

Because our database searches covered a long period, there was no need for cross-referencing. The LILACS database was concomitantly searched for articles, theses, and dissertations, articles being chosen whenever there was a duplicate entry. 

The retrieved articles were initially screened by the reading of their titles and abstracts. The inclusion criteria were as follows: having an available abstract; having been published in English, Spanish, or Portuguese; being a study of humans only; being an original, quantitative study (i.e., systematic reviews, meta-analyses, and case reports being excluded); having included individuals over 10 years of age, given that the treatment regimen recommended for children differs from that recommended for adults; having included individuals with non-multidrug-resistant pulmonary TB caused by *Mycobacterium* *tuberculosis*, including those with latent TB; having involved at least one of the drugs that constitute the treatment regimen recommended by the World Health Organization and the Brazilian National Ministry of Health (i.e., rifampin, isoniazid, pyrazinamide, and ethambutol); and having described or referred to estimators of the association between ADRs and risk factors. 

For each criterion, there were two response options: yes and no. In order to be included in the review, an article had to meet all of the inclusion criteria; that is, the response option for each of the aforementioned criteria had to be "yes". When there was uncertainty as to whether a given article met all of the inclusion criteria, the article in question was read in its entirety. A total of 85 articles (2 articles from the LILACS database and 83 articles from the MEDLINE database) were selected. Only 1 article was unavailable in full text. 

In most of the studies, statistical associations between ADRs and their risk factors had not been calculated. Therefore, the data were processed with Epi Info, version 3.5.3, ORs being calculated as measures of association between risk factors and ADRs, with 95% CIs. In addition, Pearson's chi-square test (with Yates' correction) or two-tailed Fisher's exact test was used (n < 5). Meta-analyses were performed by means of the Mantel-Haenszel test and by calculating the combined OR (cOR). For all analyses, the level of significance was set at 5%. 

## Results

A total of 1,389 articles were retrieved. Of those, 20 were retrieved from the LILACS database and 1,369 were retrieved from the MEDLINE database. There were 2 duplicate studies. The duplicates were excluded from the analysis, and 1,387 studies were evaluated. 

Eighty-four articles were selected to be read in their entirety by two independent raters, who took into account four criteria: 1) the study did include individuals over 10 years of age, given that most of the abstracts provided no such information; 2) the study examined adverse reactions to antituberculosis drugs using the terms ADR(s), side effect(s), toxicity, or adverse effect(s); 3) the study had a loss of less than 20% of the sample for the analyses of interest; and 4) the study included measures of association between ADRs and risk factors or allowed the calculation of such measures. In order to be included in the review, an article had to meet all four criteria. 

**Figure 1 -:**
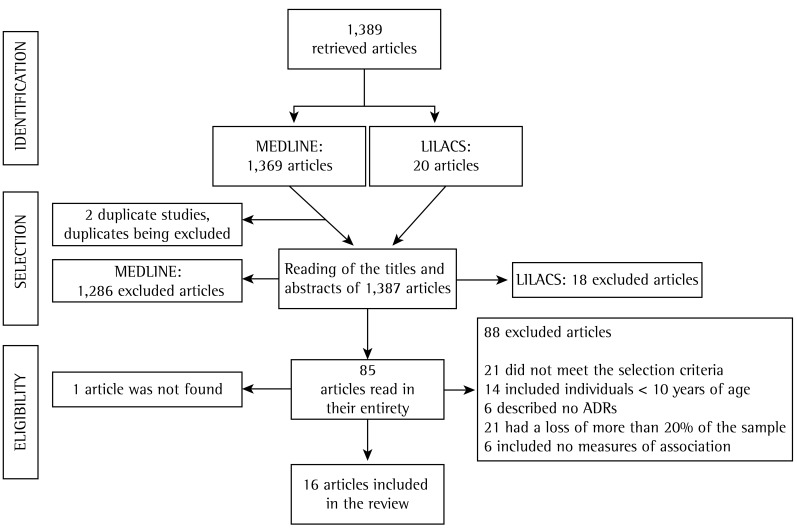
Flowchart of article selection. ADRs: adverse drug reactions.

A total of 68 articles were excluded. Of those, 21 were excluded for not meeting the inclusion criteria: much of the information that was unclear in the abstracts was clarified only after the articles were read in their entirety; 14 were excluded because they included individuals under 10 years of age; 6 were excluded for not having described any adverse reaction to antituberculosis drugs; 21 were excluded because more than 20% of the sample was lost; and 6 were excluded for not including measures of association between ADRs and risk factors or for not including data that allowed the calculation of such measures. This resulted in the inclusion of 16 articles in the present review. The articles were selected with the aid of Microsoft Office Excel 2010, and the process is illustrated in Figure 1. 

Most (63%) of the studies were conducted in Asia, 2 were conducted in Latin America, 2 were conducted in North America, 1 was conducted in Europe, and 1 was conducted in Africa. Table 1 presents the general characteristics of the studies included in the review. 

**Table 1 -:** Studies included in the systematic review, conducted in the 1965-2012 period.

Author	Study design	Age group (years)	Sample (n)	Study population	Study period	Study setting	Treatment regimen	Statistical analysis
Ai et al.^(14)^	Case-control study	≤ 60 and > 60	639	Outpatients	June of 2006 to March of 2007	China	RHZE for 2 months + RH for 4 months	Unspecified univariate and multivariate analyses
Baghaei et al.^(15)^	Population-based cohort study	< 65 and ≥ 65	761	Inpatients	January of 2006 to January of 2008	Iran	RHZE for 6 months	Χ^2^, Fisher’s exact test, Mann-Whitney test, and logistic regression
Barnes et al.^(16)^	Cohort study	≥ 15	161	Inpatients	June of 1984 to March of 1985	USA	No data	Χ^2^ and Fisher’s exact test
Cantalice Filho et al.^(17)^	Case-control study	15-49 and ≥ 60	581	Inpatients	January of 1980 to December of 1996	Brazil	RHZ for 2 months + RH for 4 months	Χ^2^ and Fisher’s exact test
Cho et al.^(18)^	Cohort study	51.2 ± 17.5 46.7 ± 18.4	132	Inpatients	June of 2004 to December of 2005	South Korea	RHZE for 2 months + HRE for 4 months	Mann-Whitney test, Χ^2^, and Fisher’s exact test
HKCS/BMRC^(19)^	Clinical trial	≥ 15	620	Unspecified	October of 1984 to October of 1986	China	SHRZ (3×/week for 6 months)	Relative frequency (%) and absolute frequency (n), unspecified univariate analysis
Kelly et al.^(20)^	Cohort study	Mean, 34.9 and 41.7	187	Inpatients	November of 1991 to May of 1993	Africa	SHRZ for 2 months/TH for 6 months	Kaplan-Meier method, unspecified univariate and multivariate analyses
Khalili et al.^(11)^	Case-control study	18-86	100	Inpatients	September of 2006 to March of 2009	Iran	RHZE for 2 months + RH for 4 months	Χ^2^
Lee et al.^(21)^	Retrospective cohort study	18-84	148 (Latent TB)	Outpatients/inpatients	April of 1999 to March of 2001	USA	RZ for 2 months	Relative frequency (%) and absolute frequency (n), relative risk, and multivariate analysis
Nanashima et al.^(12)^	Randomized cross-sectional study	22-94	100	Inpatients	May of 2005 to September of 2006	Japan	H (400 mg/day) + R (450 mg/day)	Mann-Whitney test, Χ^2^, Fisher’s exact test, and logistic regression
Martínez Sanchís et al.^(22)^	Cohort study	≥ 10 to ≥ 64	198 (Latent TB)	Inpatients	December of 1996 to December of 2002	Spain	H 300 mg/day (2 or 6 months)	Χ^2^, Fisher’s exact test, and logistic regression
Sharma et al.^(23)^	Cohort study	16-80	346	Inpatients	1996-2000	India	RHZE	Χ^2^ and logistic regression
Sirinak et al.^(24)^	Cohort study	≥ 18	769	Inpatients	May of 2005 to September of 2006	Thailand	RHZE	Unspecified univariate analysis and logistic regression
Teixeira et al.^(25)^	Case-control study	> 18	167	Inpatients	1998-2008	Brazil	H (400 mg/day) + others	Mann-Whitney test, Χ^2^, Fisher’s exact test, Student’s t-test, etc.
Teleman et al.^(26)^	Retrospective cohort study	16-82	783	Outpatients	January of 1998 to December of 1998	Singapore	RHZ for 9 months + E or S	Mann-Whitney test, Χ^2^, Fisher’s exact test, and logistic regression
No authors listed^(13)^	Clinical trial	≥ 12	908	Outpatients	Unspecified	India	R3/R5/Z5	Relative frequency (%) and absolute frequency (n), unspecified univariate analysis

TB: tuberculosis; HKCS: Hong Kong Chest Service; BMRC: British Medical Research Council; R: rifampin; H: isoniazid; Z: pyrazinamide; E: ethambutol; S: streptomycin; T: thiocetazone; R3: rifampin-streptomycin-isoniazid-pyrazinamide daily for 3 months; R5: rifampin-streptomycin-isoniazid-pyrazinamide daily for 3 months, followed by streptomycinisoniazid- pyrazinamide twice a week for 2 months; Z5: streptomycin-isoniazid-pyrazinamide daily for 3 months, followed by streptomycin-isoniazid-pyrazinamide twice a week for 2 months; and Χ2: chi-square test.

Of the 16 studies included in the present review, 56% had been conducted in the past 10 years, and 4 had been conducted in the 1980s or 1990s. All of the studies were available in English. In addition, 63% had a longitudinal design, and only 2 were clinical trials. 

The sample sizes were measured for the proposed designs. The smallest sample consisted of 100 individuals,^(^ 11 ^,^ 12 ^)^ and the largest consisted of 908 individuals.^(^ 13 ^)^

Two studies involved individuals with latent TB receiving chemoprophylaxis. The treatment given to individuals with active TB varied across studies in terms of drugs, doses, frequency, and duration. 

The age of the participants varied widely across studies, being unspecified in most.^(^ 14 ^-^ 20 ^)^ Approximately 70% of the studies involved inpatients,^(^ 11 ^,^ 14 ^-^ 18 ^,^ 20 ^-^ 25 ^)^ others involved outpatients,^(^ 21 ^,^ 26 ^)^ and 1 involved an unspecified population.^(^ 19 ^)^

Statistical analysis revealed that the risk factors associated with adverse reactions to antituberculosis drugs were as follows: gender; race/ethnicity; nationality; age; weight; marital status; treatment regimen; genetic factors; anemia; co-infection with HIV, HBV, or HCV; diabetes; liver disease; hypoalbuminemia; hyponatremia; lymphopenia; and alcohol, tobacco, or illicit drug use. 

Table 2 presents the risk factors that were significantly associated with ADRs. The ADRs were divided into gastrointestinal ADRs, neurological ADRs, immune-mediated ADRs, hepatic ADRs, and other ADRs. 

**Table 2 -:** Statistically significant associations between risk factors and adverse effects of antituberculosis drugs in the reviewed studies, conducted in the 1965-2013 period.

Author	Risk factor	ADR		OR	95% CI	Χ^2^ (Yates’ correction) or two-tailed Fisher’s exact test	Value of p
Kelly et al.^(20)^	HIV	Diarrhea +	Diarrhea −	2.63	1.17-6.37	5.461	0.019
	Present	42	83				
	Absent	10	52				
Barnes et al.^(16)^	Hyponatremia	Fever +	Fever −	16.6	3.95-146.80	Two-tailed Fisher’s exact test	0.000
	Present	9	22				
	Absent	5	51				
Barnes et al.^(16)^	Hypoalbuminemia	Fever +	Fever −	6.11	2.74-13.68	23.545	0.000
	Present	88	24				
	Absent	18	30				
Barnes et al.^(16)^	Alcoholism	Fever < 7 days	Fever > 7 days	5.22	1.30-24.76	Two-tailed Fisher’s exact test	0.014
	Present	10	23				
	Absent	4	48				
Barnes et al.^(16)^	Anemia	Fever < 7 days	Fever > 7 days	∞	3.3804 to ∞	Two-tailed Fisher’s exact test	0.000
	Present	14	54				
	Absent	0	59				
Barnes et al.^(16)^	Hyponatremia	Fever < 7 days	Fever > 7 days	4.17	1.09-17.46	4.577	0.032
	Present	9	22				
	Absent	5	51				
Barnes et al.^(16)^	Hypoalbuminemia	Fever < 7 days	Fever > 7 days	∞	1.03 to ∞	Two-tailed Fisher’s exact test	0.034
	Present	14	54				
	Absent	0	19				
Kelly et al.^(20)^	HIV	Fever +	Fever −	2.59	1.31-5.19	7.850	0.005
	Present	69	56				
	Absent	20	42				
Kelly et al.^(20)^	HIV	Oral candidiasis +	Oral candidiasis −	10.93	1.64-461.62	Two-tailed Fisher’s exact test	0.004
	Present	19	106				
	Absent	1	61				
Kelly et al.^(20)^	HIV	Kaposi’s sarcoma +	Kaposi’s sarcoma −	∞	1.15 to ∞	Two-tailed Fisher’s exact test	0.032
	Present	10	115				
	Absent	0	62				
Ai et al.^(14)^	Age	ADR +	ADR −	0.61	0.41-0.91	6.136	0.013
	< 60 years	209	288				
	> 60 years	77	65				
Lee et al.^(21)^	Recent infection	Hepatotoxicity +	Hepatotoxicity −	13.39	1.89-577.38	Two-tailed Fisher’s exact test	0.002
	Present	13	66				
	Absent	1	68				
No authors listed^(13)^	Therapeutic regimen	Jaundice +	Jaundice −	0.06	0.03-0.09	190.15	0.000
	R3	18	279				
	R5 and Z5	328	283				
No authors listed^(13)^	Therapeutic regimen	Jaundice +	Jaundice −	0.08	0.05-0.13	170.83	0.000
	R5	26	281				
	R3 and Z5	320	281				
No authors listed^(13)^	Therapeutic regimen	Jaundice +	Jaundice −	0.01	0.00-0.02	Two-tailed Fisher’s exact test	0.000
	Z5	2	302				
	R3 and R5	344	260				
Cho et al.^(18)^	NAT2	Hepatotoxicity +	Hepatotoxicity −	0.18	0.05-0.68	7.977	0.005
	Rapid or intermediate acetylator	11	102				
	Slow acetylator	7	12				
Khalili et al.^(11)^	NAT2	Hepatotoxicity +	Hepatotoxicity −	0.09	0.02-0.46	10.322	0.001
	Rapid or intermediate acetylator	5	31				
	Slow acetylator	9	5				
Nanashima et al.^(12)^	C/C genotype at rs2070401 in BACH1	Drug-induced hepatotoxicity +	Drug-induced hepatotoxicity −	9.73	2.04-90.86	Two-tailed Fisher’s exact test	0.001
	G/A or A/A genotype at rs4720833 in MAFK						
	Present	16	37				
	Absent	2	45				
Teixeira et al.^(25)^	NAT2 genotype	Hepatitis +	Hepatitis −	2.71	1.03-7.65	4.084	0.043
	Slow acetylator	18	64				
	Others	8	77				
Baghaei et al.^(15)^	Age	Hepatitis +	Hepatitis −	0.6	0.39-0.94	5.013	0.025
	< 65 years	50	416				
	≥ 65 years	49	246				
Teleman et al.^(26)^	Age	Drug-induced hepatotoxicity +	Drug-induced hepatotoxicity −	0.04	0.00-0.17	37.264	0.000
	≤ 60 years	26	613				
	> 60 years	29	368				
No authors listed^(13)^	Therapeutic regimen	Arthralgia +	Arthralgia −	0.66	0.49-0.88	8.164	0.004
	R3	137	160				
	R5 and Z5	345	266				
No authors listed^(13)^	Therapeutic regimen	Arthralgia +	Arthralgia −	0.55	0.41-0.74	17.158	0.000
	R5	133	174				
	R3 and Z5	349	252				
No authors listed^(13)^	Therapeutic regimen	Arthralgia +	Arthralgia −	2.85	2.11-3.87	49.89	0.000
	Z5	212	92				
	R3 and R5	270	334				

ADR: adverse drug reaction; Χ2: chi-square test; R3: rifampin-streptomycin-isoniazid-pyrazinamide daily for 3 months; R5: rifampin-streptomycin-isoniazid-pyrazinamide daily for 3 months, followed by streptomycin-isoniazid-pyrazinamide twice a week for 2 months; Z5: streptomycin-isoniazid-pyrazinamide daily for 3 months, followed by streptomycinisoniazid- pyrazinamide twice a week for 2 months; and NAT2: N-acetyltransferase 2.

Gastrointestinal ADRs included nausea, vomiting, indigestion, diarrhea, and other, unspecified, reactions. Co-infection with HIV was the only risk factor for gastrointestinal ADRs, with a significant association with the development of diarrhea.^(^ 20 ^)^

Neurological ADRs included vertigo and other, unspecified, reactions. However, no risk factors were significantly associated with neurological ADRs. 

Immune-mediated ADRs included fever, herpes zoster, Kaposi's sarcoma, oral candidiasis, immune reconstitution inflammatory syndrome, and other, unspecified, reactions. Co-infection with HIV was found to be a risk factor for Kaposi's sarcoma and oral candidiasis.^(^ 20 ^)^ Fever was a common ADR, being significantly associated with the following risk factors: anemia; hypoalbuminemia; hyponatremia; alcoholism; and co-infection with HIV.^(^ 16 ^)^

Hepatic ADRs were the most investigated ADRs, including jaundice, hepatitis, hepatotoxicity, and drug-induced hepatotoxicity. The rifampin-streptomycin-isoniazid-pyrazinamide combination taken daily for 3 months; the rifampin-streptomycin-isoniazid-pyrazinamide taken daily for 3 months, followed by the streptomycin-isoniazid-pyrazinamide combination taken twice a week for 2 months; and the streptomycin-isoniazid-pyrazinamide combination taken daily for 3 months, followed by the streptomycin-isoniazid-pyrazinamide combination taken twice a week for 2 months were compared and were found to be statistically significant protective factors for jaundice.^(^ 13 ^)^ In addition, primary chemoprophylaxis was found to be a statistically significant protective factor for hepatotoxicity.^(^ 21 ^)^

The association between genetic factors and hepatic ADRs was evaluated in 5 studies. A slow N-acetyltransferase 2 (NAT2) acetylator phenotype was found to be a significant risk factor for hepatitis.^(^ 25 ^)^ In contrast, a rapid/intermediate NAT2 acetylator phenotype was found to be a protective factor for hepatotoxicity.^(^ 11 ^,^ 18 ^)^ In addition, a C/C genotype at rs2070401 in BACH1 and a G/A or A/A genotype at rs4720833 in MAFK were found to be risk factors for drug-induced hepatotoxicity.^(^ 12 ^)^

Only 3 studies showed a statistically significant association between sociodemographic factors and hepatic ADRs. One study showed that being under 65 years of age is a protective factor for hepatitis,^(^ 15 ^)^ whereas another showed that being ≤ 60 years of age is a protective factor for drug-induced hepatotoxicity.^(^ 26 ^)^ Being male was found to be a significant protective factor for hepatotoxicity.^(^ 21 ^)^

Other ADRs included arthralgia, exanthema, and unspecified reactions (kidney disorders, jaundice, hearing loss, liver problems, and skin rash). One study showed that age (< 60 years) was a significant protective factor for unspecified reactions.^(^ 14 ^)^ The rifampin-streptomycin-isoniazid-pyrazinamide combination taken daily for 3 months and the rifampin-streptomycin-isoniazid-pyrazinamide taken daily for 3 months, followed by the streptomycin-isoniazid-pyrazinamide combination taken twice a week for 2 months were protective factors for arthralgia; in contrast, the streptomycin-isoniazid-pyrazinamide combination taken daily for 3 months, followed by the streptomycin-isoniazid-pyrazinamide combination taken twice a week for 2 months was a risk factor for arthralgia.^(^ 13 ^)^

**Figure 2 -:**
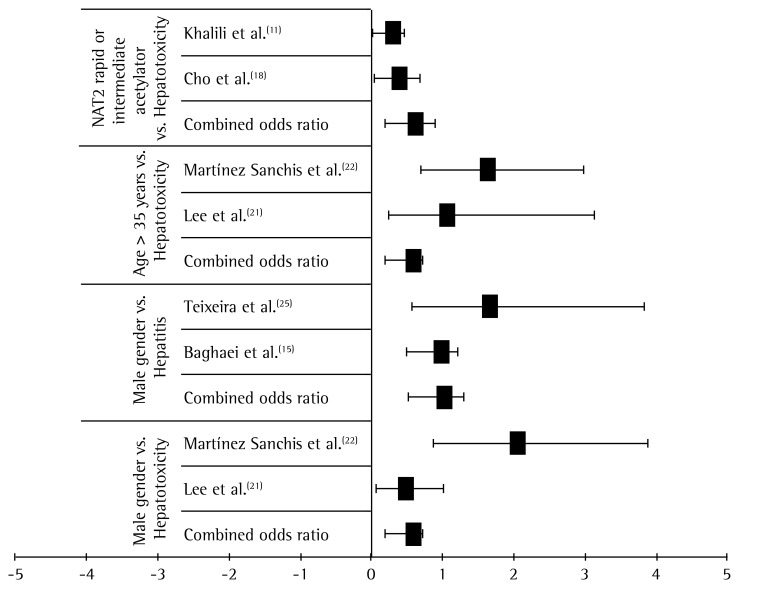
Meta-analysis of the factors associated with hepatic adverse reactions to antituberculosis drugs.

The results of our meta-analyses (Figure 2) show that protective factors for hepatic ADRs include showing a rapid/intermediate NAT2 acetylator phenotype (cOR = 0.41; 95% CI: 0.18-0.90), being 35 years of age or older (cOR = 0.38; 95% CI: 0.20-0.72), and being male (cOR = 0.38; 95% CI: 0.20-0.72). 

## Discussion

In the present review, one study^(^ 13 ^)^ demonstrated that a therapeutic regimen without rifampin was a risk factor for arthralgia. This finding suggests that rifampin can indirectly offer protection against arthralgia. 

Joint pain is considered to be a minor side effect, and, when unrelated to hyperuricemia, it is frequently associated with pyrazinamide and, more rarely, isoniazid.^(^ 8 ^)^ This is probably due to pyrazinoic acid, which is the major metabolite of pyrazinamide; pyrazinoic acid inhibits renal tubular secretion of uric acid, thus increasing its serum concentration and causing joint pain.^(^ 7 ^)^ In a multicenter study,^(^ 27 ^)^ arthralgia was reported in 6 of 617 patients receiving rifampin, isoniazid, and pyrazinamide but in none of the 445 patients who received rifampin and isoniazid. However, most (87.5%) of the studies included in the present review involved different treatment regimens. Therefore, the results obtained by combining these studies can be misleading, and this is a limitation of our review. 

The present review included 2 studies addressing latent TB rather than active TB. One of the studies^(^ 22 ^)^ showed that primary chemoprophylaxis (the treatment of predisposed individuals in order to prevent TB infection, i.e., before they present with positive tuberculin skin test results) with 300 mg of isoniazid and 50 mg of pyridoxine for 2 months was a significant protective factor for hepatotoxicity when compared with secondary chemoprophylaxis (the treatment of latent TB infection, i.e., the treatment of patients with positive tuberculin skin test results without disease), which lasted longer (i.e., 6 months). 

The duration of drug exposure can be a determinant of hepatic ADRs, given that longer exposure to toxic metabolites translates to a greater chance of severe injury. A study conducted in 2000 in Barcelona, Spain, showed that the duration of chemoprophylaxis was associated with toxic effects; however, it was impossible to establish a relationship with the type of drug used.^(^ 28 ^)^

There is divergence across studies regarding the association between HIV co-infection and ADRs during TB treatment.^(^ 10 ^)^ In patients with TB/HIV co-infection, ADRs are generally related to the immune system itself^(^ 29 ^)^ and are due to immunosuppression and drug metabolism pathways, which often generate toxic compounds. Therefore, the findings of one study,^(^ 20 ^)^ in which HIV co-infection was considered a risk factor for diarrhea, are justifiable. 

The interaction among antituberculosis drugs can potentiate their toxic effects on the gastrointestinal and hepatic systems.^(^ 22 ^)^ Breen et al.^(^ 30 ^)^ found no difference between groups of patients with and without HIV co-infection in terms of the incidence of hepatotoxicity. In another study,^(^ 29 ^)^ co-infection with HIV was found to be a risk factor for grade I hepatotoxicity, defined as a three-fold increase in the lower limit of normal for alanine aminotransferase. 

With regard to the use of alcohol, much of the alcohol ingested by humans is metabolized in the liver by the enzyme alcohol dehydrogenase. This enzyme converts alcohol to acetaldehyde, which has toxic effects even at reduced concentrations. ^(^ 31 ^,^ 32 ^)^ There is also evidence that the induction of cytochrome P450 2E1 by ethanol is related to the pathogenesis of alcoholic liver disease. ^(^ 33 ^)^ Coca^(^ 29 ^)^ reported that alcoholism is a risk factor for hepatotoxicity. However, none of the studies examining alcohol use as a risk factor for hepatotoxicity showed a significant association between the two. 

Being male was found to be significantly associated with hepatotoxicity, being a protective factor rather than a risk factor.^(^ 21 ^)^ This is possibly due to the fact that androgen activity increases (induces) hepatic microsomal enzyme activity, which allows males to metabolize drugs more effectively.^(^ 31 ^,^ 34 ^)^ However, further studies on gender-dependent variations in drug metabolism are needed in order to draw more definitive conclusions. 

With regard to genetic factors, there was a significant association between a slow NAT2 acetylator phenotype-NAT2 being the principal enzyme responsible for metabolizing isoniazid-and hepatotoxicity,^(^ 11 ^,^ 18 ^)^ given that this phenotype can generate more hepatotoxic metabolites.^(^ 31 ^,^ 35 ^)^

There is a possibility of information bias as a result of combining results of studies evaluating hepatotoxicity during antituberculosis treatment, given that several studies employ no criteria for determining the severity of hepatotoxicity, which can be classified as grade I, II, III, or IV hepatotoxicity on the basis of transaminase levels.^(^ 29 ^)^ One limitation of the present study is that most of the reviewed studies presented no information regarding the diagnostic criteria for hepatotoxicity. 

Gastrointestinal ADRs are the most common ADRs during TB treatment.^(^ 8 ^)^ They might be due to the chemical effects of the antibacterial agents, which have an effect not only on bacterial cells but also on human cells.^(^ 31 ^,^ 36 ^)^ They can therefore cause tissue damage in the central nervous system, peripheral nervous system, liver, and hematopoietic system.^(^ 37 ^)^ However, in the present review, most of the risk factors for gastrointestinal ADRs were not significantly associated with such reactions. 

Many (37.5%) of the reviewed studies evaluated the age of the participants; however, there were differences across studies in terms of age characterization, children and elderly individuals being evaluated. According to two studies,^(^ 14 ^,^ 15 ^)^ elderly patients (over 60 or 65 years of age) are more likely to have ADRs. This is due to the fact that elderly individuals have a slower metabolism, which is due to reduced enzymatic activity, reduced hepatic clearance, and reduced availability of essential endogenous cofactors. ^(^ 31 ^,^ 34 ^)^ However, in one of the aforementioned studies,^(^ 14 ^)^ the fact that the ADRs observed in the participants were not separately categorized limits this analysis. Another limitation is related to the results of two studies.^(^ 21 ^,^ 22 ^)^ Although the combination of results was numerically significant in the meta-analysis, showing that being 35 years of age or older is a protective factor for hepatic ADRs in patients receiving TB treatment, it should be evaluated with caution, given that the authors' decision not to divide the participants into different population groups (e.g., elderly and non-elderly patients) is a confounding factor in the analysis of the results. The authors' decision was based on the small sample size, adults over 50 years of age constituting less than 15% of the sample. 

The studies included in the present review were conducted in the 1986-2012 period, which is an extended period of time. The major drawback of early studies (i.e., those conducted in the 1980s or in the 1990s) was their lack of methodological rigor, with little or no information regarding sample size calculation, losses to follow-up, independent variables, statistical methods, ethical issues, and study population characteristics. Another problem is that, for none of the reviewed studies, the year of publication corresponded to the year in which the study had been conducted. Among researchers who study publication bias, some consider that the time elapsed between the completion of a study and its publication is an important factor and is related to results without statistical significance.^(^ 10 ^)^

Most of the studies included in the present review were observational studies. Only 2 clinical trials were included.^(^ 13 ^,^ 19 ^)^ Observational studies are important because of their exploratory nature, which allows inferences to be made. However, a randomized clinical trial is the most appropriate study design to evaluate the safety profile of a given drug.^(^ 36 ^,^ 38 ^)^

Given that the incidence of TB is higher among poor individuals, we expected to find, among the studies included in the present review, at least one in which socioeconomic factors, such as income and occupation, had been evaluated. However, none of the reviewed studies examined socioeconomic variables. 

The highest TB incidence rates are concentrated in African countries. However, India, China, and Indonesia together accounted for more than 40% of all TB cases in 2006.^(^ 39 ^)^ This explains why many (44%) of the studies included in the present review were conducted in the aforementioned countries. 

The relationship between TB and socioeconomic indicators appears to be associated with the level of spatial aggregation and the particular characteristics of geographic areas.^(^ 40 ^)^ In a study conducted in London, UK, it was found that for each 1% increase in the proportion of households with more than one person per room, the average TB notification rate increased by 12%.^(^ 41 ^)^ However, the association between adverse reactions to antituberculosis drugs and socioeconomic factors has yet to be confirmed. 

There is, however, an association between a low income and malnutrition, the latter being related to physiological changes. A low protein diet (poor nutrition) is related to changes in T-cell-mediated immune function, increasing susceptibility to *M*. *tuberculosis* infection and diseases.^(^ 40 ^)^ This explains the results of one study,^(^ 16 ^)^ in which albumin, iron, and sodium deficiency was a risk factor for fever, as was the use of alcohol. 

ADRs are more closely monitored in a hospital setting, where complaints and symptoms are continuously monitored. In addition, the hospital setting allows fewer losses to follow-up and more detailed data collection in longitudinal studies. ^(^ 13 ^)^ In a study conducted in the outpatient clinic of a teaching hospital in the city of São Paulo, Brazil, the frequency of minor adverse reactions was 41.1% and that of major adverse reactions was 12.8%.^(^ 7 ^)^ According to the authors of the study, the difference between their findings and those of other studies (in which the incidence was lower) might be due to the fact that patients treated at the outpatient clinic of the medical teaching hospital are routinely screened for all possible side effects. In the present review, most of the study populations consisted of inpatients. However, in several countries, the primary treatment regimens are delivered on an outpatient basis.^(^ 8 ^)^

It can be assumed that ADRs are underreported because of the difficulty in identifying such reactions and the difficulty in monitoring patients undergoing treatment. This limits the generalization of the results of the present study. Although patients are instructed to seek medical attention at a primary care clinic should any symptom arise, they rarely do in cases of mild ADRs. In addition, there is a long interval between medical visits, and ADRs are not usually reported or treated properly. 

Another limitation of the present review is related to our methodological rigor; articles that did not meet all of the inclusion criteria were excluded from the review. In other reviews, rating scales are used in order to assist in the evaluation of the studies; therefore, rather than being excluded for not meeting methodological and statistical criteria, studies simply receive low ratings on these items.^(^ 42 ^)^

Although some of the risk factors analyzed in the present review were found to be significantly associated with ADRs, most of the results did not allow us to establish correlations, given that outcomes and exposure were defined differently across studies and therefore constituted an obstacle to our meta-analysis. 

## Final considerations

In the present review, ADRs were significantly associated with age, gender, treatment regimen, alcoholism, HIV co-infection, genetic factors, and nutritional deficiencies. Individual factors such as showing a rapid/intermediate NAT2 acetylator phenotype, being 35 years of age or older, and being male are protective factors for hepatic ADRs in patients receiving antituberculosis treatment. The remaining results should be interpreted cautiously, given that most of the data collected precluded a meta-analysis and, consequently, an evaluation of heterogeneity and external validity. The present systematic review can guide future studies aimed at achieving TB control for the benefit of public health. 
